# **β**-Elemene-Attenuated Tumor Angiogenesis by Targeting Notch-1 in Gastric Cancer Stem-Like Cells

**DOI:** 10.1155/2013/268468

**Published:** 2013-04-24

**Authors:** Bing Yan, Yuqi Zhou, Shouhan Feng, Can Lv, Lijuan Xiu, Yingcheng Zhang, Jun Shi, Yongjin Li, Pinkang Wei, Zhifeng Qin

**Affiliations:** Department of Traditional Chinese Medicine, Changzheng Hospital, The Second Military Medical University, Shanghai 200003, China

## Abstract

Emerging evidence suggests that cancer stem cells are involved in tumor angiogenesis. The Notch signaling pathway is one of the most important regulators of these processes. **β**-Elemene, a naturally occurring compound extracted from *Curcumae Radix*, has been used as an antitumor drug for various cancers in China. However, its underlying mechanism in the treatment of gastric cancer remains largely unknown. Here, we report that CD44+ gastric cancer stem-like cells (GCSCs) showed enhanced proliferation capacity compared to their CD44− counterparts, and this proliferation was accompanied by the high expression of Notch-1 (*in vitro*). These cells were also more superior in spheroid colony formation (*in vitro*) and tumorigenicity (*in vivo*) and positively associated with microvessel density (*in vivo*). **β**-Elemene was demonstrated to effectively inhibit the viability of GCSCs in a dose-dependent manner, most likely by suppressing Notch-1 (*in vitro*). **β**-Elemene also contributed to growth suppression and attenuated the angiogenesis capacity of these cells (*in vivo*) most likely by interfering with the expression of Notch-1 but not with Dll4. Our findings indicated that GCSCs play an important role in tumor angiogenesis, and Notch-1 is one of the most likely mediators involved in these processes. **β**-Elemene was effective at attenuating angiogenesis by targeting the GCSCs, which could be regarded as a potential mechanism for its efficacy in gastric cancer management in the future.

## 1. Introduction

Tumor angiogenesis has long been known to have an essential role in tumor development and metastasis [1]. Although the underlying mechanisms of this process are not currently completely understood, they have conventionally been described as sequential activation involving endothelial sprouting from preexisting vessels, endothelial proliferation, migration, and differentiation to form tubes [2]. However, recent studies in cancer stem cells (CSCs) provided additional insight into tumor angiogenesis [3], as accumulating data have indicated that CSCs are involved in tumor angiogenesis not only by enhanced capacity of vascular endothelial growth factor (VEGF) secretion compared to its counterparts [4], but, more strikingly, they are strongly proangiogenic and can generate “CSC-derived endothelial progenitor cells” [5, 6]. CSC involvement in tumor angiogenesis has been reported in various cancers including brain tumors, skin tumors, and breast cancer [7–9]. Based on these results, some investigators have speculated that this phenomenon may not be uncommon in other CSC models [10]. Gastric cancer stem-like cells (GCSCs) have been identified in gastric cancer cell lines and primary tumors that are positive for the CD44 marker [11–14], which is a universal CSC marker that has been detected in many cancers, including head and neck squamous cell carcinoma, breast cancer, and prostate cancer [15, 16]. Although the role of GCSCs in tumor angiogenesis remains obscure, in other solid tumors, such as ovarian cancer, purified CD44 positive cells (also known as ovarian CSCs) have been demonstrated to possess a similar endothelial potential [17].

Notch signaling is an evolutionarily conserved signaling pathway that plays a fundamental role in embryonic development and adulthood. To date, four vertebrate Notch receptors (Notch 1–4) have been identified. In addition, five ligands, Dll1, Dll3, Dll4, and Jag1-2, have been found in mammals with multiple associated target genes including Hes, Myc, and p21 [18]. It was believed that Notch signaling plays a critical role in CSCs and was regarded as a new cancer drug target [19]. Recent studies indicated that Notch signaling plays an essential role in vascular development and angiogenesis with several promising targets including Notch-1, Dll4, and Jag1 [2, 20, 21]. In gastric cancer, a previous study indicated that the activation of Notch-1 promotes disease progression [22], and the expression of Notch-1 was significantly higher in cancer cells than in normal tissues [23]. In addition, it was reported that oxaliplatin-resistant gastric cancer cell lines showed increased levels of Jag1 and Dll4, which are associated with a higher angiogenic rate [24]. 

**Figure 1:**
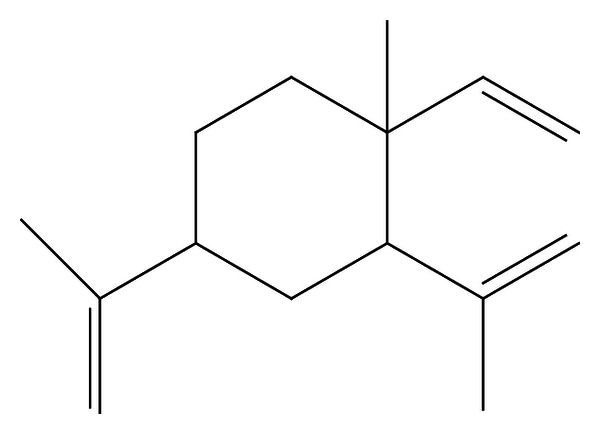
Chemical structure of *β*-Elemene.

*β*-Elemene (1-methyl-1-vinyl-2,4-diisopropenyl-cyclohexane; Figure 1), a naturally occurring compound isolated from the traditional Chinese medicinal (TCM) herb *Curcumae Radix* (Chinese Pharmacopoeia, 2010 version, http://drugs.yaojia.org/), has been approved by the State Food and Drug Administration of China to treat various solid tumors [25] and is currently under consideration for clinical studies in the United States [26]. However, the underlying mechanisms associated with its efficacy in gastric cancer are largely unknown. In this study, we investigated the role of GCSCs in tumor angiogenesis and the possible mechanism of *β*-Elemene for its efficacy.

## 2. Materials and Methods

### 2.1. Animals

Sixty-two 6- to 8-week-old male nude mice, weighing 20–24 g, were purchased from Shanghai Institute of Materia Medica (Chinese Academy of Science) and maintained under standard pathogen-free conditions (Laboratory Animal Center, Second Military Medical University). All mice were handled according to the recommendations of the National Institutes of Health Guidelines for the Care and Use of Laboratory Animals. The experimental protocol was approved by the Shanghai Medical Experimental Animal Care Commission. 

### 2.2. Cell Culture and Xenografts

**Figure 2:**
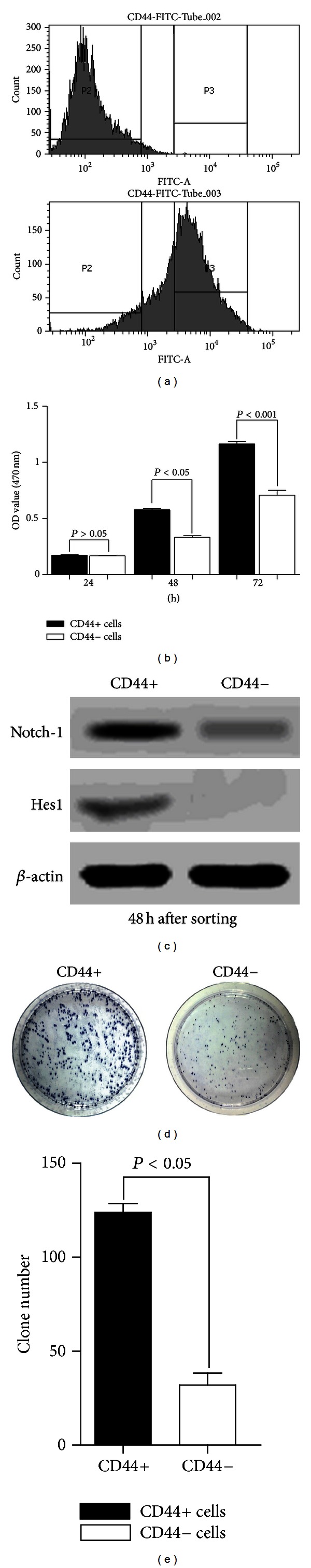
(a) FACS sorting results of cultured MKN-45 cells. (b) MTT assay revealed an enhanced proliferation capacity of CD44+ cells compared to CD44− cells 48 and 72 hours after sorting (*P* < 0.05 and *P* < 0.001, resp.). (c) Western blot of the cells 48 h after FACS sorting, which indicates a remarkable difference in Notch-1 and Hes1 expressions between CD44+ and CD44− cells. (d) Spheroid colony formation by CD44+ and CD44− cells. (e) Quantitive analysis of the spheroid clone formation efficacy of CD44+ and CD44− cells.

The human gastric cancer cell line MKN-45 was purchased from Shanghai Cells Center, the Chinese Academy of Sciences and cultured in RPMI-1640 supplemented with 10% fetal bovine serum (FBS), 10 mM HEPES, and 1% penicillin-streptomycin. Cell sorting was performed as described by Takaishi et al. [11]. In brief, confluent cells were washed once with phosphate-buffered saline (PBS) and then dissociated from plates using trypsin-EDTA and centrifuged. The cell pellets were resuspended and incubated for 30 min at 37°C with a 100-fold dilution of anti-CD44-fluorescein isothiocyanate rat monoclonal antibody (BD Biosciences, CA, USA). The samples were then stained with 4,6-diamidino-2-phenylindole (final concentration of 2 ng/mL) and then sorted with a FACSAria II flow cytometer (BD FACSAria II, CA, USA). The results were analyzed using FACSDiva version 6.1.3 software (Figure 2(a)). The purity of the sorted cells was estimated to be more than 97%. After sorting, a portion of the cells (CD44+ and CD44−) was suspended in sterile RPMI-1640 supplemented with 10% FBS, and the rest were injected subcutaneously into the axilla of the nude mice (1 × 10^5^ cells per site) using a handmade glass micropipette. For spheroid colony formation analysis, single-cell suspension of CD44+ and CD44− cells was prepared by thoroughly dissociating with 0.25% trypsin in 0.02% EDTA (Sigma, USA). The cells were then plated in culture dishes at a density of 2 × 10^2^ cells/dishes in RPMI-1640 containing 10% FBS and incubated for 2 weeks at 37°C; colonies containing more than 50 cells were counted after Giemsa staining.

### 2.3. Cell Viability and Proliferation Assay

Cell viability assays were conducted using a 3-(4,5-dimethylthiazol-2-yl)-2,5-diphenyltetrazolium bromide (MTT) kit (Beyotime, China). FACS-sorted CD44+ and CD44− cells were incubated in 96-well plates (5 × 10^4^ cells/well). The next day, the cells were treated with various concentrations of *β*-elemene (0–200 *μ*g/mL) for 48 h or for 72 h, with 6 replicates of each treatment. After incubation, 20 *μ*L of MTT reagent was added to each well (5 mg/mL), and the cells were then incubated for another 3 h at 37°C. Cell viability was determined by measuring the optical density (OD) at 470 nm with a microplate reader (Bio-Rad Laboratories, USA). The following formula was used: cell viability = (OD of the experimental sample/OD of the control group) × 100%. Another two groups that contained CD44+ and CD44− cells were assessed for cell proliferation without any intervening measurements. 

### 2.4. Drug Preparation and Administration

*β*-Elemene (97% purity) was obtained from JinGang Pharmaceuticals (Dalian, China). The mice were randomly divided into 8 groups as follows: model CD44+ group (CD44+; *N* = 10), Model CD44− group (CD44−; *N* = 10), and low-, medium- and high-dose *β*-elemene group (corresponding to 25 mg/kg, 50 mg/kg and 100 mg/kg, resp.). The low-, medium- and high-dose *β*-elemene groups included 6 mice in each of the CD44+ groups and 8 in each CD44− groups. All *β*-elemene-treated groups in the *in vitro* studies were CD44+; however, the cells used in the *in vivo* study contained both CD44+ and CD44− cells. Mice in the model groups were administered 0.4 mL 0.9% sodium chloride via intraperitoneal injection once every 2 days, while the experimental groups synchronously received the scheduled dose of *β*-elemene. Treatment was started 3 days after cell injection and continued for 8 consecutive weeks. At the end of the 8th week, the mice were euthanized, and the tumors were carefully removed and measured.

### 2.5. Immunohistochemical Staining (IHC) for CD34 and CD44

Tumor tissues were fixed in 10% formalin, embedded in paraffin, and processed using standard histological methods. Serial sections (5 *μ*m) were cut from each selected paraffin block. IHC was performed with avidin-biotin-peroxidase complex kits according to the manufacturer's instructions (Invitrogen, USA). Anti-CD44 (1 : 200, Abcam, USA) and anti-CD34 (1 : 200, Abcam, USA) antibodies were used. Primary antibodies were incubated at room temperature overnight in a humidified chamber. The positive areas in the field were counted by the Image-Pro-Express system (Olympus, Japan) at a magnification of 400x (BX51, Olympus, Japan), the final value of the IHC was calculated from eight randomly selected fields of each section by following formula: total value = (positive areas × mean OD value) × 100%.

### 2.6. Microvessel Density (MVD)

In previous studies, CD34 was demonstrated to be one of the most useful markers to identify blood vessels in gastric cancer [27–31]. To measure microvessel density (MVD) in our study, quantitative vessel counts were performed using the method described by Weidner and assessed by international consensus [32].

### 2.7. Western Blot Analysis of Notch-1, Hes1 (*In Vitro* and* In Vivo*) and Dll4, CD44 (*In Vivo*)

For our *in vitro* study, cells were washed twice with ice-cold PBS, solubilized in 1% Triton lysis buffer on ice, and then quantified using the Lowry method [33]. Cell lysate proteins (40 *μ*g) were separated by sodium dodecyl sulfate-polyacrylamide gel electrophoresis and electrophoretically transferred to nitrocellulose membranes (Millipore, USA). For our *in vivo* study, the proteins were extracted from the tissues using RIPA lysis buffer containing the protease inhibitor phenylmethanesulfonyl fluoride (PMSF, Beyotime Institute of Biotechnology, China). Proteins were separated via 10% SDS-polyacrylamide gel and transferred onto PVDF membranes (Millipore, USA). The membranes were blocked with 5% milk and then incubated with primary rabbit anti-Notch-1 (dilution 1 : 500, Abcam, USA), anti-Hes1 (dilution 1 : 200, Santa, USA), anti-Dll4 (dilution 1 : 500, Abcam, USA) and anti-CD44 (dilution 1 : 500, Abcam, USA), antibodies. Membranes were then washed and incubated with the appropriate HRP-conjugated secondary antibodies for 2 h at room temperature. Proteins were detected using an ECL detection reagent. *β*-actin was used as a loading control, and all images were analyzed using NIH Image J software.

### 2.8. Real-Time Quantitative PCR Assay for Notch-1, Hes1 (*In Vitro *and* In Vivo*) and Dll4 (*In Vivo*)

Total RNA was extracted from 50 to 100 mg of tissue according to the protocol described for the BioEasy SYBR Green I Real-Time PCR Kit (Bo Ri Technology Co., Ltd., China). The primer sequences for specific gene amplification are listed in Table 1. Real-time PCR was performed according to the standard protocol for the SYBR Premix Ex TaqTM Perfect Real-Time system (Takara, China) using an ABI 7300 detector (Applied Biosystems, USA). Fold changes in gene expression were calculated using the 2^−ΔΔCt^ method. The ODs of the target genes were compared with that of GAPDH.

### 2.9. Statistical Analysis

All data were analyzed using SPSS 18.0 software and are presented as the mean values±standard derivation. Comparisons between different groups were evaluated using a one-way ANOVA followed by the Bonferroni test. Values of *P* < 0.05 were considered statistically significant. All the data represent the mean value determined by two experienced investigators who were blinded to the design.

## 3. Results

### 3.1. GCSCs Were More Proliferative and Tumorigenic Than the CD44− Cells *In Vitro*

As shown in Figure 2(b), GCSCs showed greater proliferation than their counterparts 48 h and 72 h after sorting. The expression of Notch-1 and Hes1 was also higher in CD44+ mice than in CD44− mice 48 h after sorting, which indicated that these parameters are positively associated with the proliferation of GCSCs (Figure 2(c)). In previous study, the development of tumors with defined markers in immunodeficient mice was considered as the “gold standard” for identifying CSCs [34]. In our study (*in vivo*), 10/10 mice formed tumors in model CD44+ group, and 6/10 formed tumors in model CD44− group; however, in *β*-elemene-treated groups, this index was 6/6 in CD44+ groups and 6-7/8 in CD44− groups. In addition, spheroid colony formation assay, which was considered as an indicative of self-renewal ability and consistent with a CSC phenotype [11], revealed that CD44+ cells were more superior than CD44− cells (Figures 2(d)-2(e)). 

### 3.2. *β*-Elemene Inhibited the Viability of GCSCs as well as the Notch-1 and Hes1 Expressions *In Vitro*

**Figure 3:**
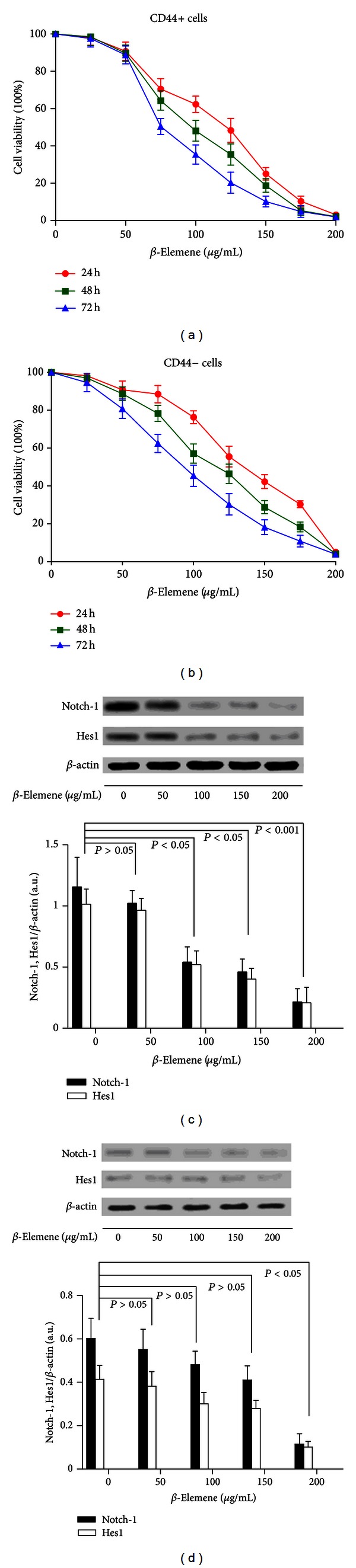
(a)-(b) Effect of *β*-elemene on the cell viability of CD44+ cells. CD44+ and CD44− cells were treated with 50, 100, 150, or 200 *μ*g/mL *β*-elemene for 24, 48, or 72 h. The cell viability was determined using an MTT assay. Dots: mean of six independent experiments; bars: SD. (c)-(d). *β*-Elemene inhibited Notch-1 and Hes1 expressions *in vitro*. CD44+ and CD44− cells were seeded in 96-well plates at a density of 5 × 10^4^ cells/well and treated with a series of *β*-elemene doses over 48 h. Cellular lysates were subjected to western blot analysis with antibodies against Notch-1, Hes1, and *β*-actin (loading control). The densitometry analysis results of the Notch-1 and Hes1 bands were normalized to *β*-actin using NIH Image J from six independent experiments. The data are expressed as arbitrary units.

Cell viability assays showed that *β*-elemene inhibited the proliferation of CD44+ cells in a dose-dependent manner (Figures 3(a)-3(b)). The IC50 values at 24, 48, and 72 h were 125.06 *μ*g/mL, 103.75 *μ*g/mL, and 72.43 *μ*g/mL for CD44+ cells and 142.61 *μ*g/mL, 117.09 *μ*g/mL, and 97.07 *μ*g/mL for CD44− cells, respectively. These data indicated that *β*-elemene has substantial antitumor effects on CD44+ MKN-45 cells. As shown in Figures 3(c)-3(d), western blotting demonstrated that *β*-elemene inhibits the expression of Notch-1 and Hes1 in CD44+ cells in a dose-dependent manner. At the low concentration of 50 *μ*g/mL, the expression of Notch-1 and Hes1 showed no variation, whereas their expression significantly decreased when the cells were treated with 200 *μ*g/mL *β*-elemene.

### 3.3. GCSCs Were Capable of Recruiting More Blood Vessels Than Were CD44− Cells, and *β*-Elemene Inhibited the Expression of CD44 and Reduced the MVD *In Vivo*

**Figure 4:**
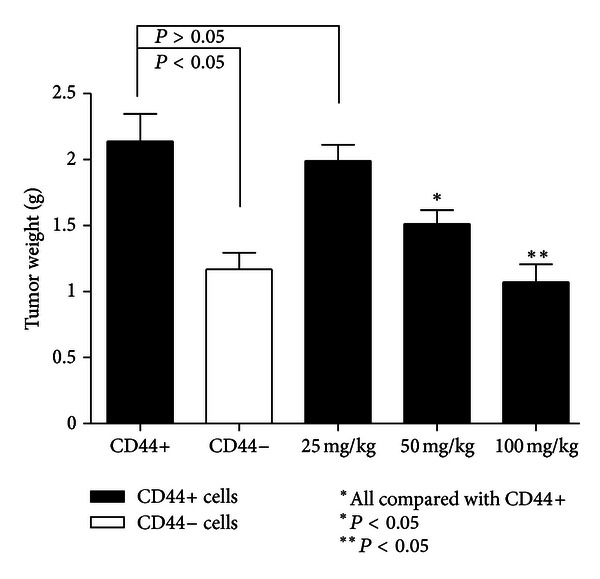
Tumor weight after 8 consecutive weeks of treatment (*N* = 6 in each groups). Model CD44+ mice had larger tumors than did model CD44− mice. *β*-Elemene treatment inhibited tumor growth in a dose-dependent manner.

**Figure 5:**
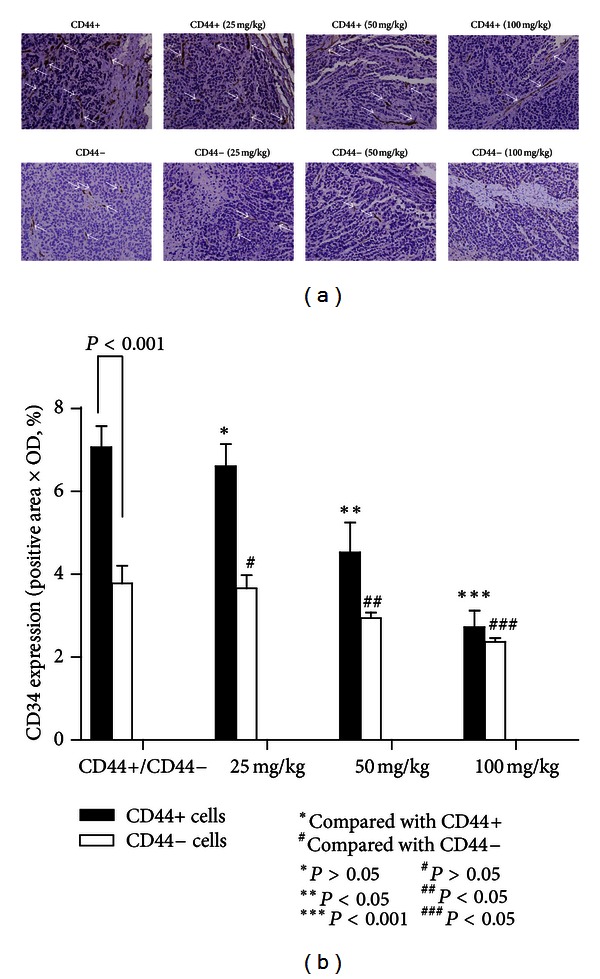
(a) Immunohistochemical staining results of the MVD. MVD was defined as a discrete CD34-positive endothelial cell aggregate, with or without definable lumina. Higher MVD could be detected in model CD44+ mice compared with model CD44− mice; *β*-elemene could inhibit the MVD in a dose-dependent manner both in the CD44+ and CD44− groups (original magnification 400x, positive areas are indicated by white arrows). (b) MVD in the different treatment groups. The model CD44+ mice showed a higher MVD than did the model CD44− mice (*N* = 6, *P* < 0.05), and *β*-elemene reduced MVD in a dose-dependent manner. Statistically significant differences in MVD could be detected in 50 mg/kg and 100 mg/kg in both the CD44+ or CD44− groups.

As shown in Figure 4, after 8 consecutive weeks of treatment, the tumor weight in model CD44+ mice was significantly higher than that in model CD44− mice (*P* < 0.05), in addition, a comparison between the *β*-elemene-treated groups and the model CD44+ group was significantly different except for the 25 mg/kg group (*P* > 0.05). Based on the IHC results (Figure 5(a)), the MVD was clearly higher in the model CD44+ group than in the model CD44− group. We detected significant differences of MVD in the 50 mg/kg and 100 mg/kg groups when compared with model CD44+ group (and model CD44− group), which indicated that *β*-elemene can inhibit angiogenesis in a dose-dependent manner (Figure 5(b)).

**Figure 6:**
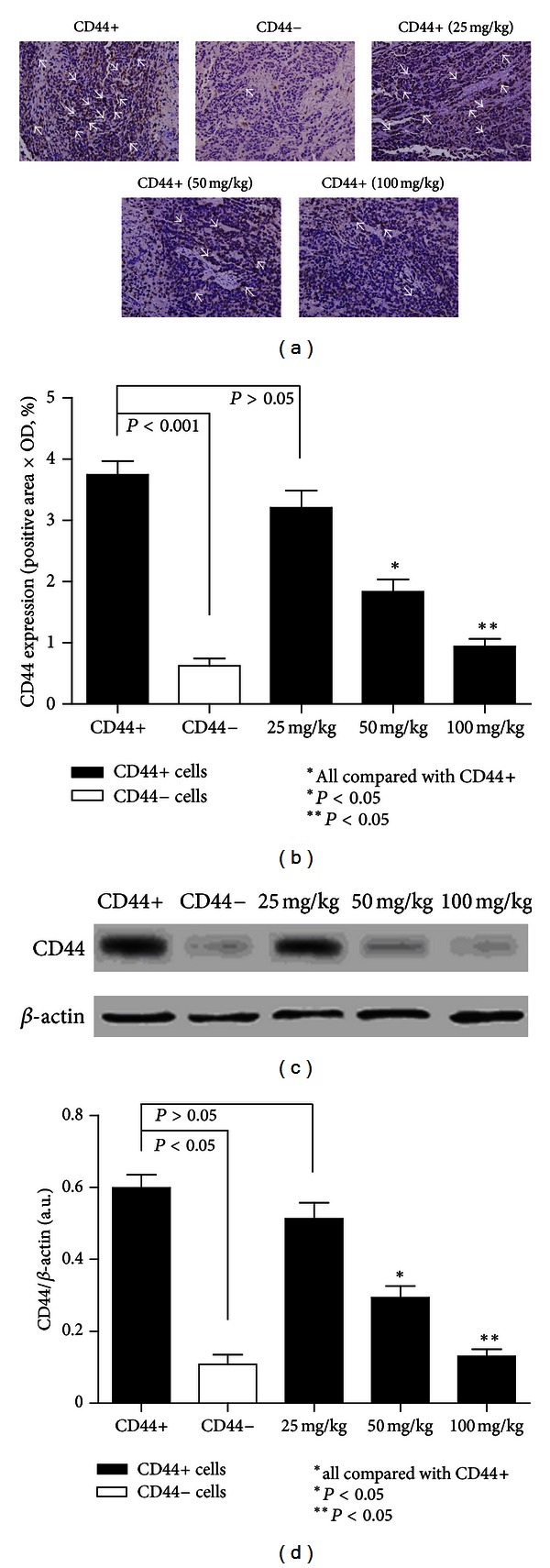
(a) Immunohistochemical staining of CD44. CD44 was highly expressed in the cancer cell membrane (original magnification 400x, positive areas are indicated by white arrows). (b) CD44 expression in each of the treatment groups (*N* = 6 in each groups). CD44 was highly expressed in model CD44+ group compared to the rest of the groups and was rarely detected in model CD44− group. The results also showed that not all of the cells in the CD44+ group were CD44 positive. *β*-Elemene inhibited the expression of CD44 in a dose-dependent manner. Statistically significant differences in expression of CD44 were also detected in the 50 mg/kg and 100 mg/kg groups (*P* < 0.05). (c)-(d) Western blot results of CD44 expression in model CD44+ group, model CD44− group, and all the *β*-elemene-treated CD44+ groups. We detected a corresponding variation of CD44 in these groups like the aforementioned IHC results of it.

In addition, as a class I transmembrane glycoprotein, CD44 was highly expressed not only in cancer cells but also in immune cells (such as leukocytes) and stromal cells (such as fibroblasts) [11]. Interestingly, despite the fact that CD44+ and all the CD44+ *β*-elemene-treated groups are GCSCs, the IHC results indicated that not all of the cells were CD44+ in these groups after the experiment, which suggested that CD44+ cells could give rise to CD44− cells (Figure 6(a)). We also detected a significant difference in CD44 expression between the model CD44+ and model CD44− groups (*P* < 0.001). In addition, in the CD44+ *β*-elemene-treated groups, the comparison of CD44+ and 50 mg/kg and CD44+ and 100 mg/kg is also significantly different in CD44 expression (*P* < 0.05) (Figure 6(b)). These results were further confirmed by western blot analysis (Figures 6(c)-6(d)).

### 3.4. Notch-1 and Hes1 Were Highly Expressed in GCSCs in Xenograft Mice, and *β*-Elemene Inhibited Their Expression in a Dose-Dependent Manner

**Figure 7:**
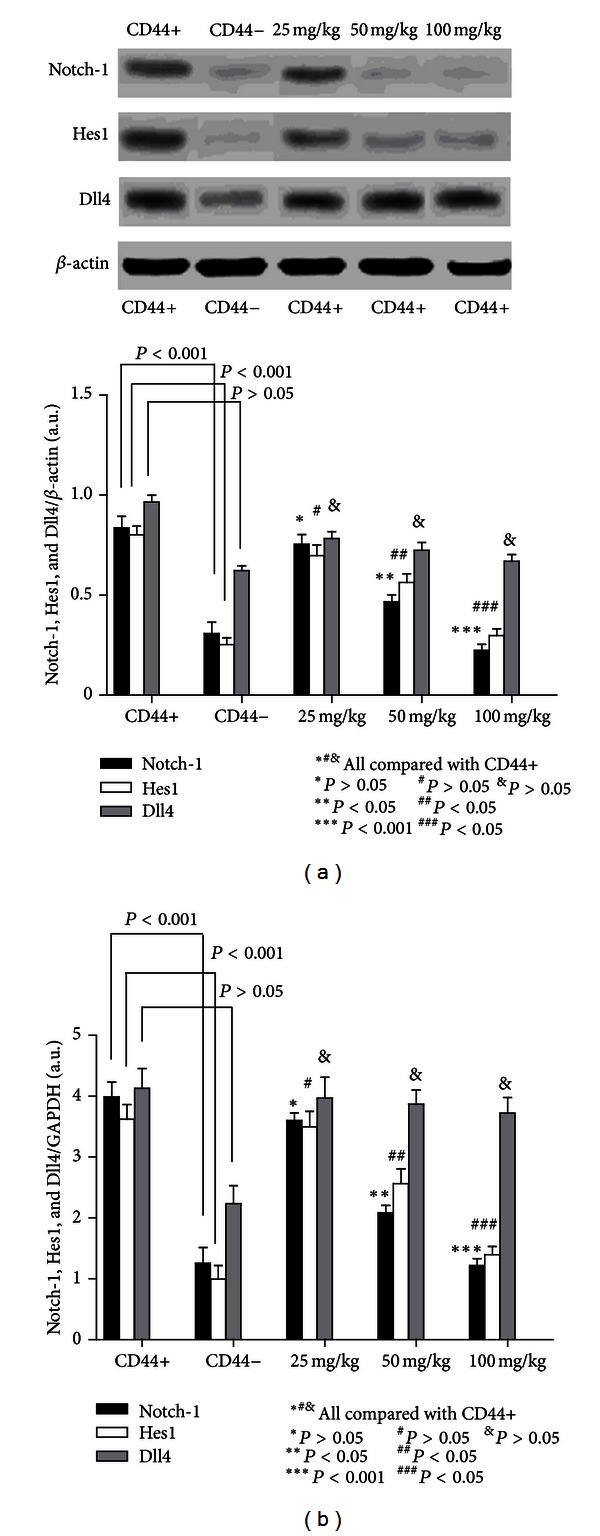
Western blot and real-time PCR analyses of Notch-1, Hes1, and Dll4 expressions *in vivo*. (a) Western blotting revealed that Notch-1 and Hes1 were highly expressed in the model CD44+ compared to the model CD44− group. *β*-Elemene inhibited the expression of Notch-1 and Hes1 in a dose-dependent manner, and statistically significant differences could be detected in the 50 mg/kg and 100 mg/kg groups. However, statistically significant differences in Dll4 expression could only be detected between the model CD44+ group and model CD44− group, and *β*-elemene failed to inhibit the expression of Dll4 in a dose-dependent manner. We found no statistically significant differences between any of the *β*-elemene-treated groups. (b) The variation of Notch-1, Hes1, and Dll4 expressions in the experimental groups was further confirmed by RT-PCR.

Notch-1 and Hes1 expressions were measured by western blot and quantitative real-time PCR. As shown in Figures 7(a)-7(b), we found that Notch-1 and Hes1 expressions were significantly increased in the model CD44+ group compared with that of the model CD44− group (*P* < 0.05). In addition, the comparison between model CD44+ group and the *β*-elemene-treated groups (except the 25 mg/kg group) also showed an obvious significant difference (*P* < 0.05). However, we failed to detect any statistically significant differences in Dll4 expression in the model CD44+ group and CD44+ *β*-elemene-treated groups, although we did observe differences in expression between the model CD44+ and model CD44− groups (*P* < 0.05). Our PCR results were in agreement with our western blotting results (Figure 7(b)).

## 4. Discussion

Accumulating data have demonstrated the pivotal role of Notch signaling in tumor angiogenesis [2]. In the present study, we observed that GCSCs have greater Notch-1 expression than the CD44− cells (*in vitro* and *in vivo*), and we also detected a higher MVD in GCSCs in xenografted nude mice. These results, together with those of the aforementioned studies [7–9], may indicate that GCSCs play an important role in tumor angiogenesis. In addition, it has been demonstrated that the downregulation of Notch-1 contributes to cell growth inhibition in various cancers [35, 36]. In our study, we found that *β*-elemene could inhibit the growth of GCSCs in a dose-dependent manner accompanied by the reduced expression of Notch-1 and Hes1. Taking into consideration that CD44 is a transcriptional target of Notch-1 [37], apparently downstream of Hes1/Hey1 [38], we concluded that *β*-elemene could attenuate tumor angiogenesis by targeting GCSCs at least in part through Notch-1 expression. Notably, although Dll4 has been demonstrated to be a key regulator in tumor angiogenesis, *β*-elemene failed to influence its expression in our study. As we know, Dll4 is an endothelium-specific ligand that is expressed at sites of vascular of normal origin [39]. Its regulation is complex and may be manipulated by multiple Notch signaling [40]. Based on our current study, it is unclear whether *β*-elemene has a limited effect on endothelial cells or other signaling pathways involved in the regulation of Dll4.

It is worth noting that naturally occurring compounds have increasingly been demonstrated to be effective in targeting CSCs. Wang et al. demonstrated that *sulforaphane*, a dietary component of broccoli/broccoli sprouts, inhibits breast CSCs [41]; Kawahara et al. observed that *quercetin*, a major polyphenol and flavonoid commonly found in many fruits and vegetables, decreases the levels of Notch-1 protein and targets pancreatic CSCs [42]. Lin et al. indicated that the *curcumin* analogue, GO-Y030, can target colon CSCs [43]. Wang et al. further reported that *curcumin* can downregulate Notch-1 [35]. Bao et al. also demonstrated that *curcumin* can attenuate CSC markers including CD44 and EpCAM [44]. Interestingly, *curcumin* (C_21_H_20_O_6_) is partially originated from the same herbal source as *β*-elemene in TCM (Chinese Pharmacopoeia, 2010 version, http://drugs.yaojia.org/). In addition, Zhen et al. suggested that arsenic trioxide (a drug derived from TCM) could deplete the cancer stem cell like population in gliomas [45]. Sun et al. further reported that arsenic trioxide may regulate the apoptosis of glioma stem cells via the downregulation Notch-1 and Hes1 [46]. Although the data concerning the ability of *β*-elemene to target CSCs is limited, the results of our study may shed light on this possibility.

The role of CSCs in tumor angiogenesis has not been fully elucidated; however, increased VEGF secretion was one of the most studied potential mechanisms, although it has not been observed in GCSCs. For example, Beck et al. demonstrated that in CD34+ skin tumors, CSCs express higher levels of VEGF than do their daughter cells [8]. Sun et al. indicated that in breast cancer, the VEGF concentration was significantly higher in breast CD44+/CD24-cell (CSCs) conditioned medium than in CD44+/CD24+ cell-conditioned medium [9]. Bao et al. found that, in comparison with matched nonstem cell-like glioma cancer populations, stem cell-like glioma cancers consistently secreted markedly elevated levels of VEGF [4]. In our study, we observed that *β*-elemene could downregulate Notch-1 and Hes1, which could result in the impaired growth of GCSCs because Notch-1 and Hes1 are involved in the self-renewal and expansion of CSCs [47–49]. Although the data concerning GCSCs and VEGF secretion are currently limited, it would be reasonable to deduce the efficacy of this pharmacologic agent, as the inhibition of the self-renewal and expansion of GCSCs reduce the blood supply.

Although the VEGF pathway has been determined to be essential for developmental angiogenesis based on a number of past studies [40], a recent study has indicated the complex crosstalk between Notch and VEGF. Briefly, it was suggested that VEGF induces Dll4/Notch signaling, while Dll4/Notch signaling modulates the VEGF pathway (especially the VEGF receptor 2) [50–52]. In a previous study, *β*-elemene was demonstrated to be effective in cancer management by multiple mechanisms, including the inhibition of MAPK/ERK and PI3K/Akt/mTOR signaling pathways [25, 53], the activation of p38 MAPK and/or JNK [54], the downregulation of survivin and hypoxia-inducible factor-1a (HIF-1*α*) [55], decreasing Bcl-2 expression [56], and inducing cell cycle arrest [57] as well as cell apoptosis [58, 59]. It is notable that many of these targets are important constituents of the upstream or downstream signaling pathway of the VEGF system. For example, Nör et al. indicated that VEGF-mediated angiogenesis is associated with the induction of Bcl-2 expression [60]. Iervolino et al. also indicated that Bcl-2 overexpression in human melanoma cells increases angiogenesis [61]. Song et al. demonstrated that HIF-1*α* enhances the expression of VEGF in gastric cancer [62]; Yoshino et al. reported that the activation of p38 MAPK contributes to increased levels of VEGF secretion in human malignant glioma cells [63]. Interestingly, some of these targets that are involved in the effective mechanism of *β*-elemene are also associated with Notch-1. For example, Wang et al. demonstrated that the downregulation of Notch-1 could be an effective approach for inhibiting cell growth, migration, and invasion, and for inducing apoptotic cell death, which is associated with the inactivation of Akt/mTOR [36]. Qi et al. indicated that Notch-1 signaling was found to downregulate the expression of cyclin and to induce the apoptosis of cancer cells through the downregulation of Bcl-2 and the activation of JNK [64]. Chen et al. observed that Notch-1 signaling facilitates survivin expression [65]. Based on these previous studies, we speculate that there may be some crosstalk between the Notch and VEGF signaling pathways that may contribute to the efficacy of *β*-elemene in our study. However, additional studies are still needed to address this assumption in the future.

## 5. Conclusion

Our study indicated that GCSCs play an important role in tumor angiogenesis, and Notch-1 is one of the most likely mediators involved in this process. *β*-Elemene was effective at attenuating angiogenesis by targeting GCSCs at least in part through Notch-1 expression. This potential mechanism for *β*-elemene may be used to manage gastric cancer in the future.

## Figures and Tables

**Table 1:** The base sequences of primers used for quantitative real-time PCR.

Primer name	Sequence
Notch-1	
Forward	CACTGTGGGCGGGTCC
Reverse	GTTGTATTGGTTCGGCACCAT
Hes1	
Forward	AGCCAACTGAAAACACCTGATT
Reverse	GGAGTTTATGATTAGCAGTGG
Dll4	
Forward	GGTGACCTGGCGAACAGACGAGCAAAAT
Reverse	GGTGACCTGGCGAACAGACGAGCAAAAT
GAPDH	
Forward	GGCATCCTGGGCTACACT
Reverse	CCACCACCCTGTTGCTGT
